# Influence of Grain Boundary Scattering on the Field-Effect Mobility of Solid-Phase Crystallized Hydrogenated Polycrystalline In_2_O_3_ (In_2_O_3_:H)

**DOI:** 10.3390/nano12172958

**Published:** 2022-08-26

**Authors:** Yusaku Magari, Wenchang Yeh, Toshiaki Ina, Mamoru Furuta

**Affiliations:** 1 Graduate School of Natural Science and Technology, Shimane University, 1060 Nishikawatsu, Matsue 690-8504, Japan; 2 Japan Synchrotron Radiation Research Institute (JASRI/SPring-8), 1-1-1 Kouto, Sayo 679-5198, Japan; 3 School of Environmental Science and Engineering and Research Institute, Kochi University of Technology, 185 Miyanokuchi, Tosayamada, Kami 782-8502, Japan

**Keywords:** polycrystalline oxide semiconductors, In_2_O_3_:H, solid-phase crystallization, thin-film transistors, high mobility

## Abstract

Hydrogenated polycrystalline In_2_O_3_ (In_2_O_3_:H) thin-film transistors (TFTs) fabricated via the low-temperature solid-phase crystallization (SPC) process with a field-effect mobility (*μ*_FE_) exceeding 100 cm^2^ V^−1^ s^−1^ are promising candidates for future electronics applications. In this study, we investigated the effects of the SPC temperature of Ar + O_2_ + H_2_-sputtered In_2_O_3_:H films on the electron transport properties of In_2_O_3_:H TFTs. The In_2_O_3_:H TFT with an SPC temperature of 300 °C exhibited the best performance, having the largest *µ*_FE_ of 139.2 cm^2^ V^−1^ s^−1^. In contrast, the *µ*_FE_ was slightly degraded with increasing SPC temperature (400 °C and higher). Extended X-ray absorption fine structure analysis revealed that the medium-range ordering in the In_2_O_3_:H network was further improved by annealing up to 600 °C, while a large amount of H_2_O was desorbed from the In_2_O_3_:H films at SPC temperatures above 400 °C, resulting in the creation of defects at grain boundaries. The threshold temperature of H_2_O desorption corresponded well with the carrier transport properties; the *µ*_FE_ of the TFTs started to deteriorate at SPC temperatures of 400 °C and higher. Thus, it was suggested that the hydrogen remaining in the film after SPC plays an important role in the passivation of electron traps, especially for grain boundaries, resulting in an enhancement of the *µ*_FE_ of In_2_O_3_:H TFTs.

## 1. Introduction

Amorphous oxide semiconductors (AOSs), represented by amorphous In–Ga–Zn–O (a-IGZO), have now become standard channel materials in thin-film transistors (TFTs) for active-matrix liquid-crystal displays and active-matrix organic light-emitting diode displays [1,2,3]. This is because a-IGZO has properties that are superior to hydrogenated amorphous Si (a-Si:H), such as a large field-effect mobility (*μ*_FE_) of over 10 cm^2^ V^−1^ s^−1^, an extremely low leakage current, a low processing temperature (<350 °C), and excellent uniformity [4,5,6,7,8,9,10,11,12]. Although the *μ*_FE_ value of a-IGZO TFTs is more than ten times higher than that of a-Si:H TFTs (<1 cm^2^ V^−1^ s^−1^), the further improvement of *μ*_FE_ values is required to expand their range of applications as alternatives to poly-Si TFTs (50–100 cm^2^ V^−1^ s^−1^) [13]. The optimization of AOS composition is one approach for improving *μ*_FE_; for example, an increase in the In ratio due the considerable spatial spread of the In 5*s* orbital with a large overlap can provide a facile electron transport path with a low electron effective mass [14,15]. Although various compositions, including In–Sn–Zn–O [16,17], In–W–Zn–O [18,19], Al–In–Sn–Zn–O [20], and In–Ga–Zn–Sn–O [21], have been proposed to enhance the *μ*_FE_, the value remains insufficiently high to compete with that of low-temperature polysilicon (LTPS) TFTs [22].

In contrast, the crystallization of OSs is an another approach for improving *μ*_FE_, because the subgap density of states originating from structural disorder and defects can be suppressed via lattice ordering. Although polycrystalline Oss, including In_2_O_3_, ZnO, and SnO_2_, have been investigated as channel materials in early oxide-based TFTs [23,24,25], they can easily create oxygen vacancies, leading to degenerate semiconductors. In addition, *µ*_FE_ degradation due to grain boundary scattering is a serious issue for polycrystalline OSs as well as poly-Si [26,27]. Polycrystalline In_2_O*_3_* films have been investigated for use as the transparent conductive oxide (TCO) in solar cells. Koida et al. reported a degenerate hydrogen-doped polycrystalline In_2_O*_3_* (In_2_O*_3_*:H) film with high electron mobility (100–130 cm^2^ V^−1^ s^−1^) produced by solid-phase crystallization (SPC) [28]. Recently, we reported a *μ*_FE_ value of 139.2 cm^2^ V^−1^ s^−1^ for a TFT obtained using hydrogenated polycrystalline In_2_O_3_ (In_2_O_3_:H) formed via SPC at 300 °C [29]. The obtained *μ*_FE_ value is comparable to the Hall mobility of single-crystalline epitaxial In_2_O_3_ films (~160 cm^2^ V^−1^ s^−1^) [30]. The as-deposited amorphous In_2_O_3_:H was converted into a polycrystalline film with lateral grain sizes of about 140 nm via SPC [29]. However, the effects of SPC temperature on the electrical and structural properties of In_2_O_3_:H films are not yet understood in detail.

In this study, we investigated the effects of SPC temperature on electron transport properties in In_2_O_3_:H films and TFTs. Hydrogen intentionally doped during sputtering was found to play an essential role in the passivation of defects, especially for the grain boundaries of the films, resulting in an enhanced *μ*_FE_ of the TFTs.

## 2. Materials and Methods

### 2.1. Fabrication of In_2_O_3_:H TFTs

In_2_O_3_:H TFTs were fabricated on a heavily doped *p*-type Si substrate with a 100 nm thick thermally grown SiO_2_ layer. The doped *p*-type Si substrate and SiO_2_ layer were used as the gate electrode and gate insulator, respectively. The 30 nm thick In_2_O_3_:H channels were deposited via pulsed direct current (DC) magnetron sputtering, without substrate heating, from a ceramic In_2_O_3_ target using a mixture of Ar, O_2_, and H_2_ gases. The gas flow ratios of O_2_ (*R*[O_2_] = O_2_/(Ar + O_2_ + H_2_)) and H_2_ (*R*[H_2_] = H_2_/(Ar + O_2_ + H_2_)) were 1% and 5%, respectively. The deposition pressure and DC power were maintained at 0.6 Pa and 50 W, respectively. The base pressure before gas introduction was below 6 × 10^−5^ Pa. After deposition, the In_2_O_3_:H films were annealed in ambient air at a temperature range of 200–600 °C for 1 h. After annealing, a 100 nm thick SiO_2_ film was deposited via reactive sputtering without substrate heating. Subsequently, Al source/drain electrodes were deposited via sputtering. Finally, the In_2_O_3_:H TFTs were annealed at 250 °C in ambient air for 1 h. In_2_O_3_:H, SiO_2_, and Al films were deposited through a shadow mask, and both the channel length and width were 300 µm.

### 2.2. Characterization Methods

The carrier concentration (*N*_e_) and Hall mobility (*µ*_FE_) of the In_2_O_3_:H films were determined by Hall effect measurements (Accent, HL5500PC) using the van der Pauw geometry at room temperature. The local structural changes of the films were evaluated through extended X-ray absorption fine structure (EXAFS) at the BL01B1 beamline in SPring-8. The In K-edge fluorescence XAFS of the films was measured using a 19-element Ge detector with an incident X-ray angle of 2° with respect to the sample surface. The XAFS data were analyzed using the Demeter software packages [31]. The macroscopic structure of the In_2_O_3_:H films was observed using electron backscattering diffraction (EBSD) (EDAX-TSL Hikari High-Speed EBSD Detector). The hydrogen concentration in the films was measured using secondary ion mass spectrometry (SIMS) (ULVAC-PHI, ADEPT-1010) with Cs^+^ as a primary ion. The chemical bonding states of the constituent elements and hydrogen were evaluated from their thermal effusion using thermal desorption spectroscopy (TDS), which was carried out while varying the stage temperature from 50 to 800 °C at a heating rate of 60 °C min^−1^. Reference films of In_2_O_3_ under similar conditions maintaining an *R*[H_2_] of 0% (without hydrogen introduction) were deposited for comparison.

## 3. Results and Discussion

### 3.1. Electrical Properties of In_2_O_3_:H Films

**Figure 1:**
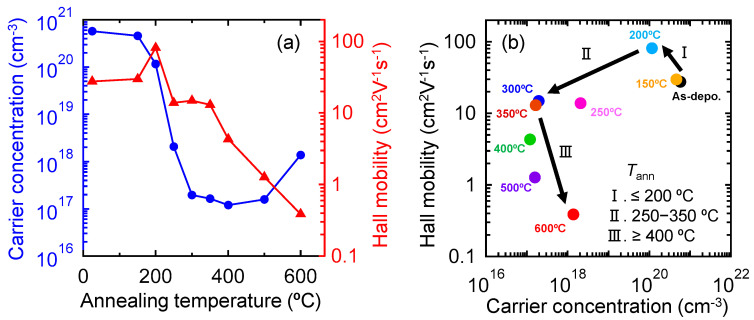
(**a**) *N*_e_ and *µ*_H_ of In_2_O_3_:H films as a function of *T*_ann_. (**b**) Relationship between *µ*_H_ and *N*_e_ for In_2_O_3_:H films with various *T*_ann_ values.

Figure 1a shows the variations in *N*_e_ and *µ*_H_ in the 50 nm thick In_2_O_3_:H films as a function of the annealing temperature (*T*_ann_). The *N*_e_ of the as-deposited film (5.7 × 10^20^ cm^−3^) began to decrease rapidly from *T*_ann_ = 200 °C, where SPC occurred. Then, the In_2_O_3_:H exhibited an almost constant *N*_e_ of ~2 × 10^17^ cm^−3^ (an appropriate value for TFT fabrication) over a *T*_ann_ range of 300–500 °C. After reaching a *T*_ann_ of 600 °C, the *N*_e_ slightly increased to 1.4 × 10^18^ cm^−3^. The *µ*_H_ of In_2_O_3_:H increased to 78.6 cm^2^ V^−1^ s^−1^ after SPC occurred (*T*_ann_ = 200 °C), whereas the *µ*_H_ of the films decreased to ~15 cm^2^ V^−1^ s^−1^ at a *T*_ann_ range of 250–350 °C. As *T*_ann_ further increased, the *µ*_H_ of In_2_O_3_:H began to decrease, resulting in a *µ*_H_ of 0.4 cm^2^ V^−1^ s^−1^ at *T*_ann_ = 600 °C.

To understand the carrier transport properties of the In_2_O_3_:H films, the relationship between *µ*_H_ and *N*_e_ for the films with various *T*_ann_ values is shown in Figure 1b. The changes in electrical properties could be classified into the following three regions: (I) enhanced *µ*_H_ at a *T*_ann_ ≤ 200 °C; (II) decreased *µ*_H_ with decreasing *N*_e_ at *T*_ann_ = 250–350 °C; and (III) decreased *µ*_H_ with constant or increasing *N*_e_ at *T*_ann_ ≥ 400 °C. The decrease in the *µ*_H_ of the polycrystalline In_2_O_3_:H films with increasing *T*_ann_ in regions II and III was considered to be due to the effects of grain boundary scattering and intragrain scattering. In general, for degenerate transparent conducting oxide materials, the mobility in the grains is determined by an optical method using the Drude model, because optical mobilities are not affected by grain boundary scattering [32,33]. However, it is difficult to determine the optical mobility of In_2_O_3_:H films annealed at *T*_ann_ ≥ 250 °C because the free electrons are significantly decreased to the order of 10^17^ cm^−3^ and the films become non-degenerate semiconductors [34,35]. Therefore, we evaluated the effects of intragrain scattering by measuring the field-effect mobility of In_2_O_3_:H TFTs. This is because when a voltage is applied to the gate, a large number of carriers (10^19^–10^20^ cm^−3^) are accumulated at the In_2_O_3_:H/gate insulator interface, and the effects of grain boundary scattering can almost be neglected since electrons tunnel through the narrow width (<1 nm) of the grain barriers at high *N*_e_ values [36].

### 3.2. Electrical Properties of In_2_O_3_:H TFTs

**Figure 2:**
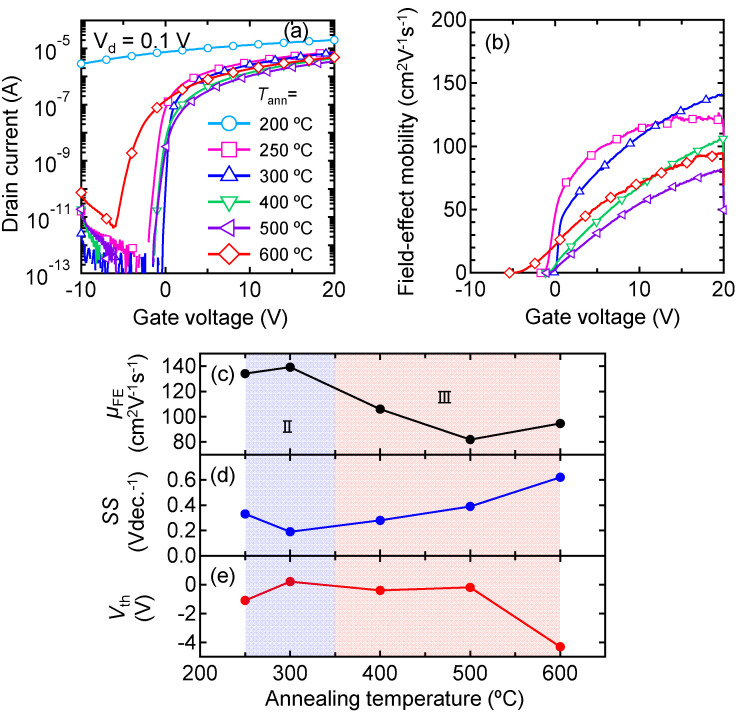
(**a**) Transfer characteristics and (**b**) *µ*_FE_ of the In_2_O_3_:H TFTs with channels annealed at various temperatures; *T*_ann_ dependence of (**c**) *µ*_FE_, (**d**) *SS*, and (**e**) *V*_th_ in the In_2_O_3_:H TFTs.

Figure 2 shows the typical characteristics of the In_2_O_3_:H TFTs annealed at *T*_ann_ values of 200–600 °C. The *µ*_FE_ was calculated from the linear transfer characteristics (*V*_ds_ = 0.1 V) using the following equation:

$$\mu_{\mathrm{FE}}=\frac{Lg_{m}}{WC_{\mathrm{ox}}V_{\mathrm{ds}}}$$

where *g*_m_ is the transconductance, *C*_ox_ is the oxide capacitance of the gate insulator, and *V*_ds_ is the drain voltage. *V*_th_ was defined by gate voltage (*V*_gs_) at a drain current (*I*_ds_) of 1 nA, and *SS* was extracted from *V*_gs_, which required an increase in the *I*_ds_ from 10 to 100 pA. The average values and standard deviations (*σ*) of the characteristics of five TFTs on the same substrate are shown in Supplementary Figure S1 and Supplementary Table S1. The In_2_O_3_:H TFT annealed at 200 °C did not exhibit any switching (conductive behavior) because the In_2_O_3_:H film was still in a degenerated state (*N*_e_ = 5.7 × 10^20^ cm^−3^). The TFTs annealed at 300 °C exhibited the best performance, with the largest field-effect mobility (*µ*_FE_) of 139.2 cm^2^ V^−1^ s^−1^ and smallest subthreshold swing (*SS*) of 0.19 Vdec^−1^ with an appropriate threshold voltage (*V*_th_) of 0.2 V (shown in Figure 2c,e). Although the *µ*_H_ of the In_2_O_3_:H films decreased to 14.9 cm^2^ V^−1^ s^−1^ with a *N*_e_ of 2.0 × 10^17^ cm^−3^ after annealing at 300 °C, as shown in Figure 1, extremely high *µ*_FE_ values were obtained from the TFTs at higher gate voltages (Figure 2b). Thus, we concluded that the decrease in *µ*_H_ in region II shown in Figure 1b) was mainly due to an increase in the potential barrier at the grain boundary caused by a decrease in *N*_e_, rather than a decrease in intragrain mobility. In contrast, the TFT characteristics were slightly degraded when *T*_ann_ was increased to 600 °C, i.e., the *µ*_FE_ decreased and the *SS* value increased. Comparing *µ*_H_ and *µ*_FE_ after *T*_ann_ = 600 °C, the *µ*_H_ significantly deteriorated to 0.4 cm^2^ V^−1^ s^−1^, whereas the *µ*_FE_ of the TFTs was maintained at 94.6 cm^2^ V^−1^ s^−1^. This result suggests that grain boundary scattering is a dominant factor that limits the *µ*_H_ in films annealed at 400 °C and higher.

### 3.3. Structural Properties of In_2_O_3_:H Films

**Figure 3:**
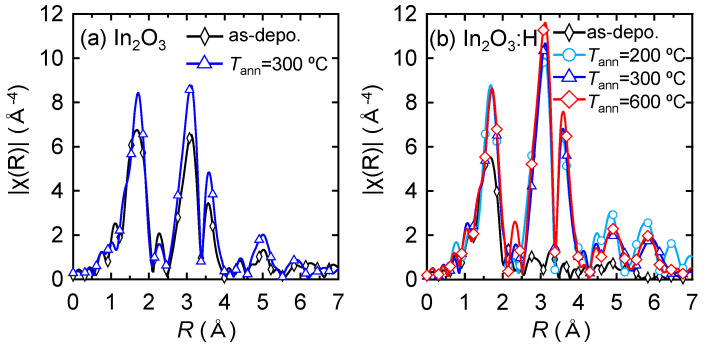
FT EXAFS spectra of the In K-edge for the (**a**) In_2_O_3_ and (**b**) In_2_O_3_:H films with various *T*_ann_ values.

To investigate the crystallinity of the films, the effect of *T*_ann_ on the local structure of the In_2_O_3_:H films was evaluated using XAFS. Figure 3 shows Fourier-transformed (FT) EXAFS spectra of the In K-edge for the (a) In_2_O_3_ and (b) In_2_O_3_:H films as a function of the phase uncorrected interatomic distance. The as-deposited In_2_O_3_ film without hydrogen introduction during sputtering exhibited three obvious peaks (Figure 3a), which corresponded to the nearest oxygen (In–O) and the second and third nearest In (In–In and In–In^*^). Using the values for the crystalline In_2_O_3_ powder standard, the interatomic distance (*R*) and Debye–Waller factor (*σ*^2^) for the films were determined. The *k*-range of the EXAFS data used in the analyses was *k* = 3–14 Å^−1^ with a *k*-weight of 3. The fitting carried out in the *R* space was from *R* = 1.0–4.0 Å for the three-shell model. As shown in Table 1, the *R*_In–O_, *R*_In–In_, and *R*_In–In*_ values of the as-deposited In_2_O_3_ film without hydrogen were 2.16, 3.35, and 3.82 Å, respectively, which agreed well with the interatomic distance of the In_2_O_3_ bixbyite structure (space group Ia3, number 206) [37,38,39]. When the In_2_O_3_ film was annealed at 300 °C, no noticeable changes in *R* were observed (Figure 3a), while the peak intensity increased in each spectrum, resulting in a decrease in *σ*^2^. This result indicates that thermal annealing improved the structural disorder of the films. By introducing hydrogen during sputtering, the second and third nearest peaks disappeared in the as-deposited In_2_O_3_:H film, while the intensity of the first nearest peak decreased (Figure 3b), resulting in an increase in *σ*^2^_In–O_ to 0.0112. In contrast, after annealing at 200 °C, the intensities of all peaks increased significantly and the intensities of the second and third nearest peaks were higher than those of the In_2_O_3_ film annealed at 300 °C. These results indicate that medium-range ordering was lost around In in the initial In_2_O_3_:H film, whereas medium-range ordering at distances equal to or longer than the second neighbor significantly improved after annealing at 200 °C. This is in agreement with a previous study using electron backscatter diffraction which confirmed that the amorphous state of the initial In_2_O_3_:H film and the grain size of the In_2_O_3_:H film were enlarged through SPC [29]. Therefore, the increase in *µ*_H_ after annealing at 200 °C, as shown in Figure 1b) (region I), is due to an increase in grain size as well as the improvement of the local structural order of the In_2_O_3_:H films. As *T*_ann_ increased from 200 to 300 °C, the intensity of the first nearest peak was constant, while the intensities of the second and third nearest peaks increased slightly, resulting in a decrease in *σ*^2^_In–In_ (0.0056) and *σ*^2^_In–In_ (0.0049). This result indicates that the medium-range ordering was improved by annealing at 300 °C, which is in good agreement with the high intragrain mobility obtained via *µ*_FE_ for the In_2_O_3_:H TFT annealed at 300 °C. As *T*_ann_ increased from 300 to 600 °C, the intensities of the second and third nearest peaks further increased, whereas *R* remained almost constant. Despite the improvement in the crystallinity of the In_2_O_3_:H films observed when annealing at 600 °C, the *µ*_FE_ of the TFTs slightly decreased to 94.6 cm^2^ V^−1^ s^−1^, as shown in Figure 2c, and the *µ*_H_ significantly deteriorated to 0.4 cm^2^ V^−1^ s^−1^. The deterioration of the *µ*_H_ of the In_2_O_3_:H films (Figure 1b, Region III) and the *µ*_FE_ of the TFTs annealed at ≥400 °C could not be explained by local structural changes in the In_2_O_3_:H films. Thus, it was suggested that the decreases in the *µ*_H_ and the *µ*_FE_ of the TFTs in region III shown in Figure 1b and Figure 2c were mainly due to the formation of defects at grain boundaries.

**Figure 4:**
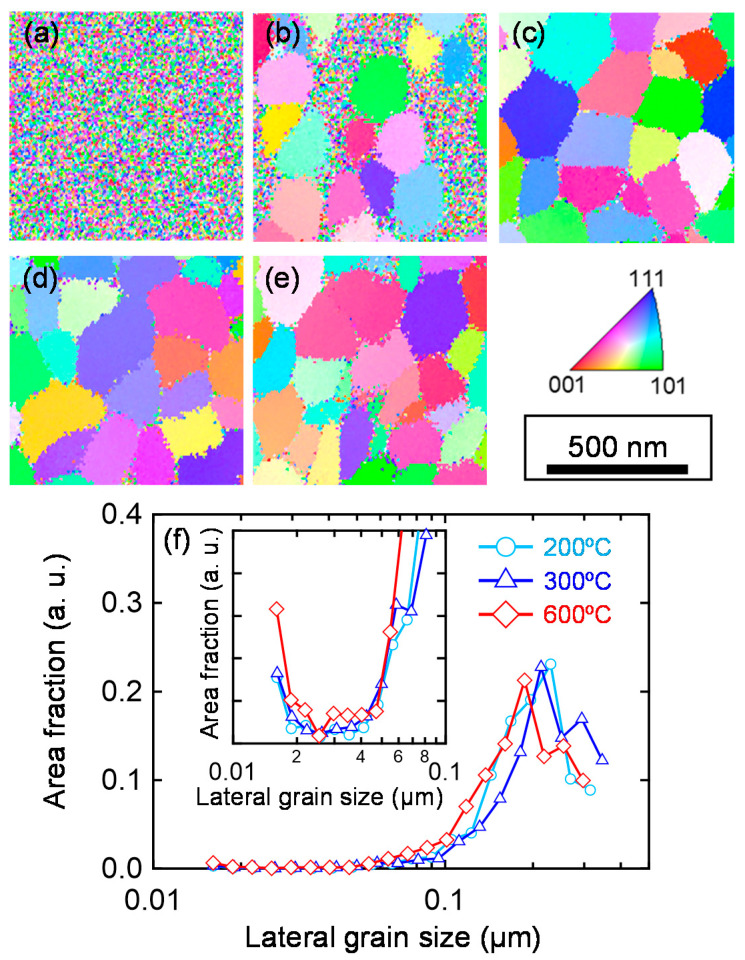
EBSD images of the (**a**) as-deposited, (**b**) 150 °C, (**c**) 200 °C, (**d**) 300 °C, and (**e**) 600 °C annealed In_2_O_3_:H films. (**f**) Area fraction of each grain size obtained from the In_2_O_3_:H films with various *T*_ann_ values. The inset shows a magnified view of the small area fraction at a small grain size.

To investigate the origin of the deterioration of the *µ*_H_ of In_2_O_3_:H films at *T*_ann_ ≥ 400 °C, we performed EBSD measurements. Figure 4a–e depicts the EBSD images along the normal direction for the In_2_O_3_:H films with various *T*_ann_ values. The as-deposited amorphous In_2_O_3_:H film was converted into a polycrystalline In_2_O_3_:H film with grain structure embedded in the amorphous matrix at a *T*_ann_ of 150 °C (Figure 4b). At a *T*_ann_ of 200 °C (Figure 4c), the film was fully crystallized with a grain size of around 200 nm. After that, no significant difference was observed in the crystal grain size with increasing *T*_ann_ values up to 600 °C. The corresponding area fractions of the crystalline phase are shown in Figure 4f. All films showed a maximum area fraction for a grain size of ~200 nm; however, a small proportion of the area fraction with a grain size of ~15 nm was increased in the In_2_O_3_:H film annealed at 600 °C, as shown in the insets of Figure 4f. Moreover, these small domains were located in between the large grains, as shown in Figure 4e. The results indicate that when the In_2_O_3_:H films were annealed at 400 °C and higher, small domains were created at grain boundaries that served as electron traps, resulting in a decrease in the *µ*_H_ in region III shown in Figure 1b.

**Figure 5:**
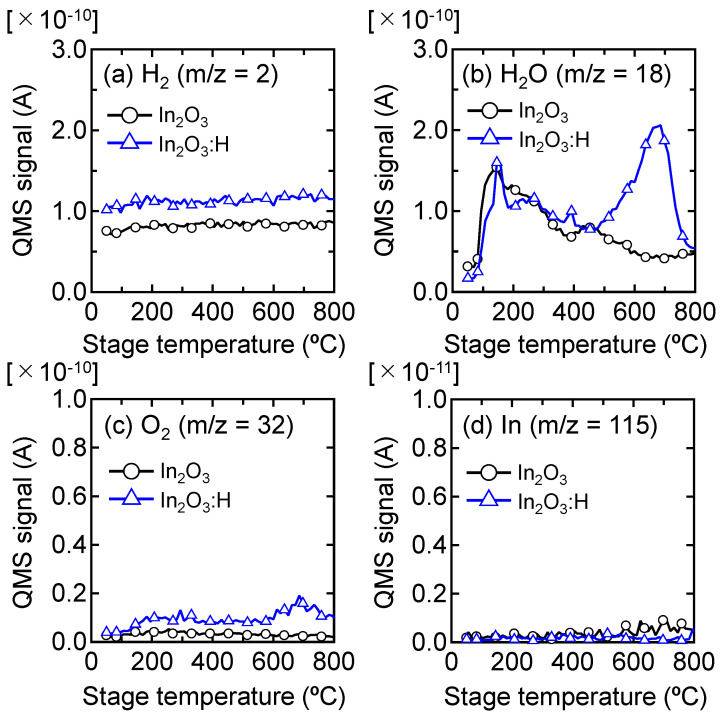
(**a**) H_2_, (**b**) H_2_O, (**c**) O_2_, and (**d**) In desorption spectra from as-deposited In_2_O_3_ and In_2_O_3_:H films.

To understand the mechanism of structural deterioration at the grain boundaries of In_2_O_3_:H films at *T*_ann_ ≥ 400 °C, we performed TDS measurements. Figure 5 shows the TDS spectra of H_2_, H_2_O, O_2_, and In for the In_2_O_3_ and In_2_O_3_:H films. We first note that H_2_, O_2_, and In desorption were negligible for both types of films, while the H_2_O desorption was high for the In_2_O_3_:H film in particular. Large amounts of H_2_O were desorbed at a stage temperature of 400–800 °C for the In_2_O_3_:H film (Figure 5b). The amount of hydrogen in the film was quantitatively evaluated using SIMS, and it was found that 2.6 × 10^21^ cm^−3^ of hydrogen remained in the film after SPC at 300 °C, which was one order of magnitude higher than that of the In_2_O_3_ film. The H_2_O desorption temperature (400 °C) for the In_2_O_3_:H film corresponded well to a *T*_ann_ of 400 °C, at which the *µ*_H_ of the films started to degrade. During annealing at a *T*_ann_ of 400 °C and higher, H or –OH inside grains may migrate to grain boundaries and react with a neighboring H at the boundary, resulting in the generation of H_2_O molecules. As a consequence, defects are formed at grain boundaries. In other words, the presence of H and/or –OH bonds in the In_2_O_3_:H film after SPC plays an important role in the passivation of defects, especially for grain boundaries. In general, the *SS* value of a TFT is strongly affected by defects near the semiconductor Fermi level [2,12]. We recently reported from hard X-ray photoelectron spectroscopy analysis that intentionally introduced hydrogen is effective in reducing defects near the Fermi level in amorphous IGZO [40,41,42,43,44]. The *SS* values of the In_2_O_3_:H TFTs, shown in Figure 2d, increased at a *T*_ann_ of 400 °C and higher, indicating defect creation. Thus, we believe that the H and/or –OH bonds remained in the films after SPC passivated the defects near the Fermi level, leading to the high *µ*_FE_ and steep *SS* values of the In_2_O_3_:H TFT annealed at 300 °C. Thus, it is worth noting that grain boundary scattering, which is a serious issue for polycrystalline Si TFTs, may not have a strong influence on the *µ*_FE_ of polycrystalline In_2_O_3_:H TFTs.

## 4. Conclusions

In summary, we investigated the effects of annealing temperature on the electron transport properties of In_2_O_3_:H films and TFTs. The changes in the electrical properties of the In_2_O_3_:H films were classified into the three following regions.

(I) When *T*_ann_ = 200 °C, *µ*_H_ increased by converting amorphous In_2_O_3_:H into a polycrystalline In_2_O_3_:H film with an increase in grain size.

(II) When *T*_ann_ = 250–350 °C, *µ*_H_ decreased with decreasing *N*_e_. However, when *µ*_H_ exhibited low values, the medium-range ordering in the grains improved. The In_2_O_3_:H TFT annealed at 300 °C exhibited the best performance, with a *µ*_FE_ of 139.2 cm^2^ V^−1^ s^−1^.

(III) When *T*_ann_ ≥ 400 °C, although *µ*_H_ significantly decreased with constant or increasing *N*_e_, the *µ*_FE_ of the TFTs was maintained at 94.6 cm^2^ V^−1^ s^−1^. The medium-range ordering of the In_2_O_3_:H network was improved by a *T*_ann_ of 600 °C, while a large amount of hydrogen was desorbed at 400–800 °C, resulting in defect creation at the grain boundaries. Thus, it was suggested that the hydrogen remaining in the film after SPC plays an important role in the passivation of electron traps, especially for the grain boundaries of the In_2_O_3_:H films, resulting in an enhancement of the *µ*_FE_. We believe that the SPC-grown In_2_O_3_:H TFTs are promising candidates for use in future electronics applications.

## Figures and Tables

**Table 1:** EXAFS fitting results for first, second, and third shells in the In_2_O_3_ and In_2_O_3_:H films.

		1st Shell (In–O)		2nd Shell (In–In)		3rd Shell (In–In^*^)	
	*T*_ann_ (°C)	*R*_In–O_ (Å)	*σ*^2^_In–O_ (Å^2^)	*R*_In–In_ (Å)	*σ*^2^_In–In_ (Å^2^)	*R*_In–In*_ (Å)	*σ*^2^_In–In*_ (Å^2^)
In_2_O_3_	as-depo.	2.16	0.0090	3.35	0.0065	3.82	0.0066
In_2_O_3_	300	2.16	0.0073	3.36	0.0062	3.83	0.0063
In_2_O_3_:H	as-depo.	2.13	0.0112	-	-	-	-
In_2_O_3_:H	200	2.16	0.0072	3.36	0.0059	3.83	0.0051
In_2_O_3_:H	300	2.17	0.0072	3.36	0.0056	3.84	0.0049
In_2_O_3_:H	600	2.17	0.0072	3.37	0.0051	3.84	0.0046

## Data Availability

The data are available on the request from corresponding author.

## Supplementary Materials

The following supporting information can be downloaded at: https://www.mdpi.com/article/10.3390/nano12172958/s1, Figure S1: Variations of transfer characteristics of the In_2_O_3_:H TFTs with channels annealed at various temperatures; Table S1: Summary of the TFT properties.
