# Supplementary material for: Influence of Grain Boundary Scattering on the Field-Effect Mobility of Solid-Phase Crystallized Hydrogenated Polycrystalline In2O3 (In2O3:H)
Source: Nanomaterials (Basel). 2022 Aug 26;12(17):2958. doi: 10.3390/nano12172958 (PMC9458122; doi:10.3390/nano12172958)
Supplement: Supplementary file 1 [file nanomaterials-12-02958-s001.zip › nanomaterials-1841994-supplementary.pdf]

nanomaterials

MDPI

Supplementary Material

Influence of Grain Boundary Scattering on Field-Effect Mobility of Solid-Phase Crystallized Hydrogenated Polycrystalline In_2O_3 (In_2O_3:H)

Yusaku Magari [1,*], Wenchang Yeh [1], Toshiaki Ina [2] and Mamoru Furuta [3]

[1] Graduate School of Natural Science and Technology, Shimane University, 1060 Nishikawatsu, Matsue 690-8504, Japan
[2] Japan Synchrotron Radiation Research Institute (JASRI/SPring-8), 1-1-1 Kouto, Sayo 679-5198, Japan
[3] School of Environmental Science and Engineering and Research Institute, Kochi University of Technology, 185 Miyanokuchi, Tosayamada, Kami 782-8502, Japan
* Correspondence: magari.yusaku@riko.shimane-u.ac.jp; Tel.: +81-852-32-8903

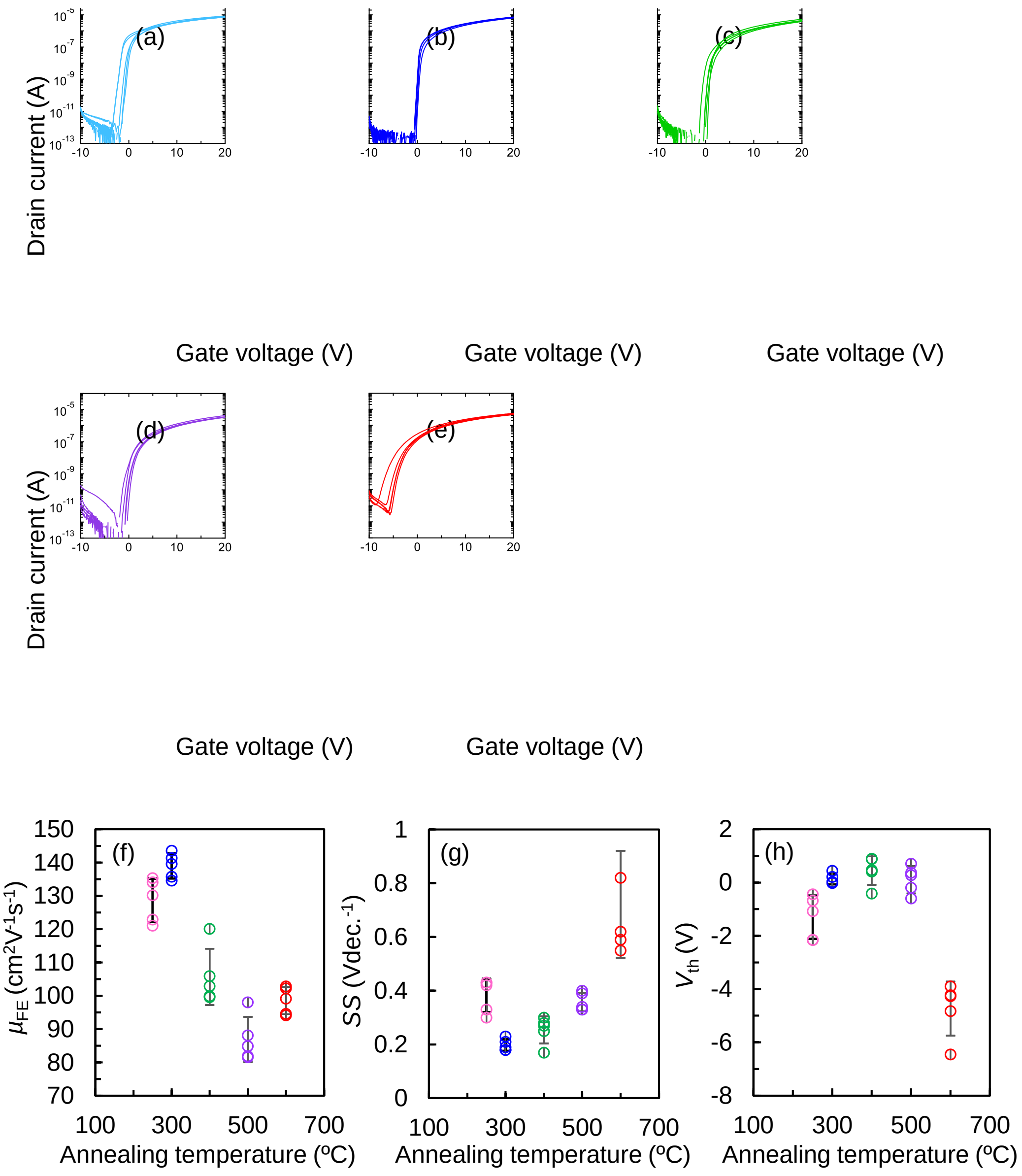

Figure S1. Variations of transfer characteristics of the In_2O_3:H TFTs with channels annealed at various temperatures; (a) 250 °C, (b) 300 °C, (c) 400 °C, (d) 500 °C, and (e) 600 °C. Five TFTs on the same substrate were measured. Variations of (f) μ_{FE}, (g) *SS*, and (h) V_{th} evaluated from five TFTs. (V_{ds} = 0.1 V, W/L = 300/300 μm)

Table S1. Summary of the TFT properties. The average values and standard deviations (*σ*) of the characteristics of 5 TFTs on the same substrate. *σ* are shown in parentheses.

Annealing temperature (°C)	μ_{FE} ($cm^2V^{-1}s^{-1}$)	*SS* ($Vdec.^{-1}$)	V_{th} (V)
250	128.7 (5.9)	0.38 (0.06)	−1.3 (0.7)
300	139.0 (3.3)	0.19 (0.02)	0.2 (0.2)
400	105.7 (7.6)	0.25 (0.04)	0.4 (0.5)
500	86.9 (6.1)	0.36 (0.03)	0.1 (0.5)
600	98.5 (3.7)	0.72 (0.18)	−4.7 (0.9)
